# Prevalence and factors associated with early discontinuation rate of Implanon utilization among women who ever used Implanon in Kucha District Gamo Gofa Zone, Southern Ethiopia

**DOI:** 10.1186/s12905-020-01096-1

**Published:** 2020-10-23

**Authors:** Mamecha Mesha, Akalewold Alemayehu, Deresse Daka

**Affiliations:** 1 College of Medicine and Health Sciences, Faculty of Health Science, Hawassa University, Hawassa, Ethiopia; 2 College of Medicine and Health Sciences, Faculty of Medicine, Hawassa University, Hawassa, Ethiopia

**Keywords:** Implanon, Discontinuation rate, Kucha, Ethiopia

## Abstract

**Background:**

The promotion of contraception in countries with high birth rates has the potential to reduce poverty, hunger, maternal, and childhood deaths. Every year in sub-Saharan Africa approximately 14 million unintended pregnancies occurred and a sizeable proportion was due to poor use of short-term hormonal methods. Contraceptive hormonal implants are highly effective and suitable for almost all women at any stage of their reproductive lives. On the other hand, early discontinuation of the Implanon contraceptive method utilization is one of the foremost problems amid the family planning program. Early discontinuation of the Implanon contraceptive method and reasons for such discontinuation lingers the most significant anxiety for family planning programs. In unindustrialized countries, contraceptive discontinuation due to health concerns is generally higher; these complaints are often related to service quality. Hence, this study aimed to assess the prevalence and factors associated with early discontinuation of Implanon among women who ever used Implanon in Kucha district, Gamo Gofa Zone, Southern Ethiopia.

**Methods:**

Implanon contraceptive device users were selected from the Kucha district using a cross-sectional community-based survey from January to March 2018. A total of 430 women were selected and data were collected through face-to-face interviews by using a pre-tested structured questionnaire. Data were cleaned, coded, and entered into Epi-Info version 7statistical software. Factors that showed association in a bivariate analysis that has *a p *value of less than 0.25 were entered into multiple logistic regression models for controlling confounding factors. The strength of statistical association was measured by adjusted odds ratio, at 95% confidence intervals, and *p *value < 0.05 were considered as statistically significant variables.

**Result:**

The result of this study revealed that the overall discontinuation rate of Implanon in the study was 34%. Variables having statistically significant association with Implanon discontinuation were women who never use a contraceptive method other than Implanon (AOR = 2.96, 95% CI 1.53–5.74), women who didn’t make discussion with a partner (AOR = 3.32, 95% CI 1.57–7.04), poor counseling and follow up (AOR = 9.23, 95% CI 4.7–18.13), fear of side effects (AOR = 0.12, 95% CI 0.058- 0.24) and poor satisfaction of service (AOR = 5.2, 95% CI 2.77- 9.76)

**Conclusion:**

The overall early discontinuation rate of Implanon in the study area was high. The main factors associated with early discontinuation of Implanon were contraceptive ever use, discussion with partner, poor follow-up of counseling, fear of side effects, and un-satisfaction by the services given during the insertion rate of Implanon.

## Background

Implants are long-acting, reversible, radiopaque, matchstick-sized, flexible, progestin-filled capsules that are placed just under the skin of the upper arm [1]. Early Implanon discontinuation is defined as discontinuation at less than two and a half years after the insertion of Implanon [2].

The promotion of contraception in countries with high birth rates has the potential to reduce poverty, hunger, maternal deaths, and childhood deaths [3]. Every year in sub-Saharan Africa approximately 14 million unintended pregnancies occurred and a sizeable proportion was due to poor use of short-term hormonal methods [4].

The most common contraception method in the world was female sterilization 19%, Intra-Uterine Contraceptive Device (IUCD) were 14% and pills were 9% among women aged 15 to 49 years who were married or in a union [5]. The Ethiopian Federal Ministry of Health (FMoH) developed a plan to expand the contraceptive method mix by providing Implanon at the community level since 2009 [6–8]. However, still there is low utilization in general, and even those who have started to use the method are discontinuing it [9].

A side effects, desire to become pregnant, menstrual disturbance, health concerns, spouse disapproval, and switching to another method are the major reasons that women discontinue using Implanon methods [10, 11].

An education, parity, side effects, and dissatisfaction of services and irregularity of menses were the major factors associated with early discontinuation of Implanon utilization among women aged between 15 and 49 years. Also, non-medical reasons such as planned pregnancy, plan to conceive in the near future and divorce, age, ethnicity, parity, socioeconomic factors were the common factor for Implanon discontinuation [10, 12, 13, 9, 14–16]. Therefore, this study aimed to assess the prevalence and factors associated with early discontinuation of Implanon among women who ever used Implanon in Kucha district, Gamo Gofa Zone, Southern Ethiopia.

## Methods

### Study area

Kucha district is one of the most densely populated districts in the Gamo Gofa zone, Southern Ethiopia. The capital of the district is Selamber which is 442 km south of Addis Abeba. The estimated total number of women in the reproductive age group (15–49) of the district was 44,091 and the coverage of Implanon utilization in 2017 was 23%.

### Study design and period

Community-based cross-sectional study design was conducted from January 2018 to March 2018.

### Study population

The study populations had been all randomly selected reproductive age group (age range of 15–49) women who are registered for Implanon use from Jan 1st, 2015 to Dec 31st, 2017 in Kucha district.

### Sampling technique

There are thirty three kebeles in the woreda. From 33 kebeles, 19 kebeles were selected by a simple random sampling method. About 430 study participants had been allocated proportionally to kebeles after the number of clients identified from the family planning registration book of the health facility for the selected kebeles which has an individual address and full information. Among 10,141 women for whom Implanon was initiated in the last 1 year, since January 1st/2015 to Dec 31st/2017 in Kucha district, 430 participants were selected. Individual study participants were selected by systematic random sampling. The first client in each kebele was selected among the numbers from 1 to Kth by a simple random sampling lottery method. Then the next study subject was selected and interviewed at every “K” interval until the sample size was fulfilled. Data has been collected from the community using a well-trained interviewer with pre administered structured questionnaire format (Additional file 1).

The information collected from each study participants was kept in a secured file without a participant’s name and another meticulous revealing. Also, it was not out in the open to anyone except the investigators. At the end of the data analysis, the questionnaire was locked within the file box. The health information and other supporting advice were given to the women who are Implanon discontinuation.

The English version of the questionnaire has been translated to local (Gammoththo) language and back translated to English to ensure its consistency by two persons who have language and medical background. The quality of the data was maintained by cross-checking daily and entered, coded, and cleaned, in Epi-Info version 7, statistical software package then transported to SPSS windows version 20. Descriptive statistics were done to assess basic client characteristics and the prevalence of early Implanon discontinuation.

Bivariate analysis using a logistic regression technique was done to see the crude association between the independent and dependent variables separately. Variables with a *p* value of less than 0.25 in the bivariate analysis had been entered into a multi-variable model as a candidate variable to determine independent predictors of early Implanon discontinuation among women who ever used Implanon by controlling confounding factors. The strength of statistical association was measured by adjusted odds ratio (AOR), at 95% confidence intervals (CI), and *p *value < 0.05 was considered as a significant variable [17].

## Results

### Socio-demographic characteristics:

A total of 430 women have participated in this study with a 100% response rate. The age of the study participants was between 18 and 41 years with a mean and SD of 28.9 ± 4.5 years. Almost all 423 (98.4%) of the respondents were married and living with their partners. About three fourth of the respondents, 321 (74.7%) were protestant, and 388 (90.2%) were Gamo in an ethnic group. The client occupation of the participants was housewives, 408 (94.9%), and three fourth of 318 (74%) their husbands were a farmer. About 308 (71.6%) of the study participants had no formal education (Table 1).

**Table 1:** Socio-demographic characteristics of Implanon users in Kucha District Gamo Gofa Zone, Southern Ethiopia March 2018 (n = 430)

Variables	Frequency (n = 430)	Percentage (%)
*Women’s age*		
≤ 30	290	67.4
≥ 31	140	32.6
*Religion*		
Orthodox	107	24.9
Protestant	321	74.7
Muslim	2	0.5
*Ethnic group*		
Gamo	388	90.2
Goffa	29	6.7
Wolayta	10	2.3
Amhara	3	0.7
*Marital status*		
Married	423	98.4
Divorced	2	0.5
Widowed	3	0.7
Single	2	0.5
*Women’s occupation*		
Housewife	408	94.9
Government employee	7	1.6
Merchant	12	2.8
Day labor	3	0.7
*Husband occupation*		
Farmer	318	74
Merchant	31	7.2
Day labor	39	9.0
Government employee	42	9.8
*Women’s education*		
No formal education	181	42.1
Read and write	127	29.5
Grade 5–8	76	17.7
Grade 9–12	33	7.7
College diploma and above	13	3.0
*Husband education*		
No formal education	104	24.2
Read and write	132	30.7
5–8 grade	113	26.3
9–12 grade	43	10
College diploma and above	38	8.8
*Family size*		
5 and below	167	38.8
6 and above	263	61.2
*Distance from health facility (km)*		
Less than 5 km	156	36.3
5 km and above	274	63.7

The information collected from each study participants was kept in a secured file without a participant’s name and another meticulous revealing. Also, it was not out in the open to anyone except the investigators. At the end of the data analysis, the questionnaire was locked within the file box. The health information and other supporting advice were given to the women who are Implanon discontinuation.

The quality of the data was maintained by cross-checking daily and entered, coded, and cleaned, in Epi-Info version 7, statistical software package then transported to SPSS windows version 20. Descriptive statistics were done to assess basic client characteristics and the prevalence of early Implanon discontinuation.

About 323 (75.1%) respondents had given birth three and above whereas 101 (23.5%) of participants given birth 1 to 2 times with a mean (± SD) number of children 4 (± 1.9) while 6 (1.4%) of the studied women were nulliparous. From the studied participants, 51 (11.9%) desire to have a child in near future.

All 430 (100%) of participants had heard about the modern contraceptive method before Implanon insertion. About 269 (62.6%) of the participant had ever used a modern contraceptive method before inserting Implanon (Table 2).

**Table 2:** Past Contraceptive utilization history and Counseling Service of Implanon users in Kucha District Gamo Gofa Zone, Southern Ethiopia March 2018 (n = 430)

Character	Response	Number	Percent
Ever heard about contraceptive methods	Yes	430	100
	No	0	0
Ever used modern contraceptive methods	Yes	269	62.6
	No	161	37.4
Perceived Satisfaction on the service provision	Yes	282	65.6
	No	148	34.4
Appointment for checkup	Yes	278	64.7
	No	152	35.3
Counseling service before insertion	Yes	422	98.1
	No	8	1.9
Follow up counseling	Yes	293	68.1
	No	137	31.9
Type of counseling	Individual	219	50.9
	With husband	113	26.3
	Mass	91	21.2
	With other relatives	7	1.6
Decision to use the method	Client	387	90.0
	Provider	43	10

**Fig. 1:**
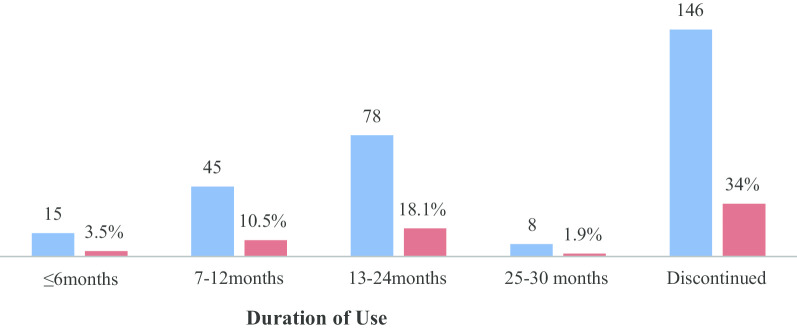
Early Implanon discontinuation rate and duration of use in Kucha District Gamo Gofa Zone, Southern Ethiopia March 2018

The continuation rate of the Implanon in the study area was 284 (66%) while; the early discontinuation rate of the Implanon utilization in the study area was 146 (34%). These women who discontinued had used Implanon for the duration of between 4 and 28 months with a mean (± SD) of 15 (± 6.6) and a median duration of 14 months use. About 14% of the study participants were used less than 24 months (Fig. 1).

### Factors associated with early Implanon discontinuation

An early discontinuation rate of Implanon among women not used the modern contraceptive method before Implanon insertion were 3 times more likely than ever used [Adjusted Odds Ratio, AOR = 2.96, (95% CI 1.5, 5.7)]. An early discontinuation rate of Implanon utilization among women who are not discussing with their partners was 3.3 times more likely than others, [AOR = 3.3 (95% CI 1.6, 7.0)] (Table 3).

**Table 3:** Factors associated with Implanon discontinuation in Kucha District, Gamo Gofa Zone, Southern Ethiopia March 2018

Variables	Discontinuation of Implanon		COR (95% CI)	AOR (95% CI)	*p* value
	Yes (%)	No (%)			
*Client age grouped*					
≤ 30	93 (32.1)	197 (67.9)	1	1	
> 30	53 (37.9)	87 (62.1)	1.290 (0.847, 1.966)	1.211 (0.599, 2.449)	0.59
*Grouped annual income in Ethiopian Birr (ETB)*					
< 10,000	72 (52.2)	66 (47.8)	1	1	
10,001–20,000	68 (27.5)	179 (72.5)	0.348 (0.225, 0.538)	0.552 (0.281, 1.084)	0.08
20,001–30,000	4 (12.1)	29 (87.9)	0.126 (0.042, 0.379)	0.190 (0.047, 1.265)	0.19
> 30,000	2 (16.7)	10 (83.3)	0.183 (0.039, 0.868)	0.060 (0.006, 1.112)	0.18
*Partner education*					
No formal education	46 (44.2)	58 (55.8)	1	1	
Read and write	43 (32.6)	89 (67.4)	0.609 (0.358, 1.036)	0.493 (0.223, 1.091)	0.08
5–8 grade	38 (33.6)	75 (66.4)	0.639 (0.369, 1.107)	0.483 (0.204, 1.143)	0.09
9–12 grade	8 (18.6)	35 (81.4)	0.288 (0.122, 0.681)	0.260 (0.071, 1.354)	0.06
Collage and above	11 (28.9)	27 (71.1)	0.514 (0.231, 1.144)	0.648 (0.193, 2.176)	0.48
*Grouped number of children*					
≤ 5	124 (36.6)	215 (63.4)	1	1	
> 5	22 (24.2)	69 (75.8)	0.553 (0.326, 0.938)	0.750 (0.334, 1.684)	0.48
*Contraceptive ever use other than Implanon*					
Yes	59 (21.9)	210 (78.1)	1	1	
No	87 (54)	74 (46)	4.185 (2.740, 6.392)	2.962 (1.529, 5.740)*	*0.001*
*Discussion with partner*					
Yes (*discussing with a partner*)	83 (24.8)	252 (75.2)	1	1	
No (*Not discussing with a partner*)	63 (66.3)	32 (33.7)	5.977 (3.653, 9.781)	3.323 (1.568, 7.043)*	*0.002*
*Method decider*					
My own decision	118 (30.5)	269 (69.5)	1	1	
Professional decision	28 (65.1)	15 (34.9)	1.255 (2.192, 8.261)	1.244 (0.430, 3.594)	0.68
*Follow-up counseling*					
Yes	45 (15.4)	248 (84.6)	1	1	
No	101 (73.7)	36 (26.3)	15.462 (9.420, 25.380)	9.229 (4.700, 18.125)*	*0.001*
*Fear of side effects*					
Yes	123 (48.8)	129 (51.2)	1	1	
No	23 (12.9)	155 (87.1)	0.156 (0.094, 0.257)	0.118 (0.058, 0.241)*	*0.001*
*Perceived service satisfaction*					
Yes	58 (20.6)	224 (79.4)	1	1	
No	88 (59.5)	60 (40.5)	5.664 (3.659, 8.770)	5.199 (2.770, 9.759)*	*0.001*

^*^Significant association in Multivariable logistic regression

Clients not getting follow-up counseling were 9.2 times more likely to early discontinuing Implanon as compared to those who got follow-up counseling service [AOR = 9.2, (95% CI 4.7, 18.1)]. Women not satisfied by the service provided during the insertion were 5.2 times more likely to discontinue Implanon as compared to who satisfied by the service provided [AOR = 5.2, (95% CI 2.8, 9.8)]. However, clients not developed side effects were 88% less likely to early discontinue Implanon as compared to clients who developed side effects [AOR = 0.22, (95% CI 0.1, 0.2)].

## Discussion

The continuation rate of the Implanon in the study area was 284 (66%). The discontinuation rate of contraceptive Implanon in the study area was 146 (34%) with a mean duration of 15 ± 6.6 months. The finding of this study is lower than the report of Debre Markos town, Northwest Ethiopia, in 2016 which was 146 (46.5%) [16]. his might be due to inadequate follow-up counseling and unexpected side effects of the method. On the other hand, the result was higher than the studies conducted in Nigeria (26.1%), Tigray region (16%), and Malaysia (22.86%) [9, 14, 15]. This might be due to the educational status of the study participants [14, 15], low male partners' involvement, and low practice of discussion with partners before the Implanon insertion [18].

Implanon as compared to women who developed side effects which is similar with the study done at Durame Town, Southern Ethiopia, Debre Markose, Northwest Ethiopia and Egypt [9, 16, 19, 20]. In this study, mothers who never used a modern contraceptive method previously were found 3 times more likely to early discontinue contraceptive Implanon than mothers who ever use it. This could be due to the fact that experienced mothers acquired the necessary knowledge and attitude towards the modern contraceptive method, while others could be influenced by false beliefs, myths, and misconceptions [13]. The other possible reasons could be fear of pregnancy delaying, side effects, and disagreement on the method used with a partner.

The probabilities of early discontinuation rates among inappropriate follow-up counseled women were 9.23 times more than those who get appropriate follow-up counseling. This finding was also supported by other studies done in Tigray, Northern Ethiopia, Debre Markos, Northwest Ethiopia [14, 16]. This is might be during follow up counseling time the women may get supplementary recommendation on side effects and supportive treatment information from service providers, consequently encouraged to continue their Implanon utilization. Moreover, those who suffer from menstrual commotion could get supportive treatment from health care providers during follow-up time, and hence they could lengthen the utilization of Implanon.

Women who were not satisfied perceptively by the service given during the insertion of Implanon were 5 times more likely to discontinue Implanon as compared to those who were satisfied by the service given during the insertion of Implanon. This is similar to the study conducted in the Tigray region, and Debre Markos town, Northwest Ethiopia [9, 16]. This is might be due to uninterested women on the method chosen. Furthermore, the confidentiality of the service, elucidation of the service supplier, communication skill, and other service provision during the insertion of Implanon may contribute to being discontinuity of Implanon.

Women who did not discuss with their partners are 3.3 times more likely to discontinue Implanon as compared to those who discussed it with male partners. This is also similar to the study conducted in Central and Northern Tigray, Ethiopia [9, 21]. This could be associated with a lack of good communication between partners.

### Limitation

This study is not a large-scaled sampled study. Moreover, recall bias was seen during data collection. This study is a quantitative method also it is another limitation.

## Conclusion

The overall early discontinuation rate of Implanon in the study area was high. The main factors associated with early discontinuation of Implanon were contraceptive ever use, discussion with partner, poor follow-up of counseling, fear of side effects, and un-satisfaction by the services given during the insertion rate of Implanon. The continuation rate of the Implanon might be increased when increasing the awareness of the utilization of Implanon, pre-service and in-service training, pre-insertion counseling, follow-up, and close monitoring is very important to women who use Implanon.

## Supplementary information

**Additional file 1.** Questionnaire and participant information sheet with consent form.

## Data Availability

The data used/ analyzed during the current study available from the corresponding author on reasonable request.

## Abbreviations

AOR: Adjusted odds ratio
COR: Crude odds ratio

## References

[1] DaVanzo J, Hale L, Razzaque A, Rahman M (2007). Effects of inter pregnancy interval and outcome of the preceding pregnancy on pregnancy outcomes in Matlab, Bangladesh. Int J Obstet Gynecol..

[2] Harvey C, Charrlotte S, Lucke J (2009). Continuation rates and reasons for removal among Implanon® users accessing two family planning clinics in Queensland, Australia. Contraception.

[3] Cleland J, Bernstein S, Ezeh A, Faundes A, Glasier A, Innis J (2006). Family planning: the unfinished Agenda. Lancet.

[4] David H, Iphigeneia M, Erin M (2008). Unintended pregnancy in sub-Saharan Africa: magnitude of the problem and potential role of contraceptive implants to alleviate it. Contraception.

[5] Population Division of the Department of Economic and Social Affairs of the United Nations Secretariat (2013). World contraceptive patterns.

[6] Asnake M, Solter C, Tilahun Y, Vespia M. Strengthening health systems to ensure equitable access to implant removal services in Ethiopia. 2013.

[7] Asnake M, Tilahun Y. Scaling up community-based service delivery of Implanon: the Integrated Family Health Programâ s experience training health extension workers. 2010. http://www2.pathfinder.org/site/DocServer/LAFP.pdf?docID=18321.

[8] Asnake M, Henry EG, Tilahun Y, Oliveras E. Addressing unmet need for long-acting family planning in Ethiopia: uptake of single-rod progestogen contraceptive implants (Implanon) and characteristics of users. Int J Gynaecol Obstet. 2013;123 Suppl 1:e29–32. 10.1016/j.ijgo.2013.07.003. 10.1016/j.ijgo.2013.07.003 24035007

[9] Birhane K, Hagos S, Fantahun M. Early discontinuation of implanon and its associated factors among women who ever used implanon in Ofla District, Tigray, Northern Ethiopia. Int J Pharma Sci Res: IJPSR. 2015;6(3):544–51.

[10] Mastor A, Khaing SL, Omar SZ (2011). Users’ perspectives on implanon in Malaysia, a multicultural Asian country. Open Access J Contracept.

[11] Teunissen AM, Grimm B (2013). Continuation rates of the subdermal contraceptive implanon (R) and associated influencing factors. Eur J Contracept Reprod Health Care.

[12] Burusie A (2013). Reasons for premature removal of Implanon among users in Arsi Zone, Oromia Region, Ethiopia. Open Access J.

[13] Chaovisitsaree S (2005). One year study of Implanon on the adverse events and discontinuation. J Med Assoc Thai.

[14] Madden T, Eisenberg DL, Zhao Q, Buckel C, Secura GM, Peipert JF (2012). Continuation of the etonogestrel implant in women undergoing immediate postabortion placement. Obstet Gynecol.

[15] Mastor A, Khaingsi L, Omar SZ (2011). Users’ perspectives on implanon in Malaysia, a multicultural Asian country. Open Access J Contracept.

[16] Siyoum M, Mulaw Z, Abuhay M, Kebebe H (2017). Implanon discontinuation rate and associated factors among women who ever used implanon in the last three years in Debre Markos Twon, North Ethiopia. ARC J Public Health Community Med.

[17] Nageso A, Gebretsadik A (2018). Discontinuation rate of Implanon and its associated factors among women who ever used Implanon in Dale District, Southern Ethiopia. BMC Women's Health.

[18] Mrwebi KP, Ter Goon D, Owolabi EO, Adeniyi OV, Seekoe E, Ajayi AI (2018). Reasons for discontinuation of Implanon among users in Buffalo City Metropolitan Municipality, South Africa: a cross-sectional study. Afr J Reprod Health.

[19] Abdel-Razik M, Said M (2012). Implanon use pattern among ministry of health and population clients 2008–2012.

[20] Tamrie YE, Gebre E, Mesele H, Argaw D (2015). Determinants of long acting reversible contraception method use among mothers in extended postpartum period, Durame Town, Southern Ethiopia: a cross sectional community based survey. J Health Res.

[21] Gebremedhin M, Tesfaye G, Belachew A, Desta D (2016). Factors influencing modern contraceptive method preference among women of reproductive age in central zone of Tigray Region, Northern Ethiopia. Int J Healthc.

