# Supplementary material for: Prevalence and factors associated with early discontinuation rate of Implanon utilization among women who ever used Implanon in Kucha District Gamo Gofa Zone, Southern Ethiopia
Source: BMC Womens Health. 2020 Oct 23;20:239. doi: 10.1186/s12905-020-01096-1 (PMC7583223; doi:10.1186/s12905-020-01096-1)
Supplement: Supplementary file 1 — Additional file 1. Questionnaire and participant information sheet with consent form. [file 12905_2020_1096_MOESM1_ESM.docx]

# ANNEX- 1: Participant information sheet and consent form

**Hawassa University, College of Medicine and Health Sciences, School of Public and Environmental Health Department**

**A. Participant information sheet and consent form**

**Title**: Assessment prevalence and risk factors associated with early discontinuation of implanon among women who ever used implanon in kucha district, Gamo Gofa Zone, Southern Ethiopia, 2018

My name is. -----------------------------------------------I temporarily represent Hawassa University, college of Medicine and health science, School of Public and Environmental Health. The research is purely for the academic purpose. This is a study to be conducted with the objective of assessing prevalence and risk factors associated with early discontinuation of implanon among women who ever used implanon in kucha district. As this study is directly related to women who use implanon, you are one of the women who have been selected randomly to participate in this study. Participation to this study is strictly voluntarily and anonymity will be respected. You are required to fill this questionnaire with the options that best represent your response and your responses will be kept confidential and there will be no way of linking your individual responses to the final results of the study findings. We would like to inform you that the responses that you provide to the questions are very essential, not only, for the successful accomplishment of the study, but also for producing relevant information which will be helpful in the planning and implementation of intervention activities to prevent early discontinuation of implanon.

I hope that you will participate in this study and provide correct information to all the questions.

Are you willing to participate in the interview? Yes___________ (continue the interview). No___________ (Thank and stop)

(Signature of the interviewer certifies that consent will be obtained verbally.)

Interviewer name _________________Sign ___________Date of interview ____________

Supervisor name___________________ Sign_____ __ Date of supervision_______________

# ANNEX-2: Questionnaires

1. Kebele----------------------- 2. Questionnaire code --------------------

**Part One; Respondents Socio-demographic information**

| S.No | Questions | Alternative /choice of response | code | Skip |
| --- | --- | --- | --- | --- |
| 101 | What was your age at time of implanon insertion? | …………… |  |  |
| 102 | What is your educational status? | 1. Cannot read and write…….1  2. 0-4 grade (Read and write)..2  3. 5-8 grade………………….3  4. 9-12 grade………………..4  5. College diploma and above..5 |  |  |
| 103 | To which religion do you belong? | 1. Orthodox……………1  2. Protestant…………….2  3. Muslim……………….3  4. Other, specify----- |  |  |
| 104 | To which ethnic group do you belong? | 1. Gamo………………….1  2. Gofa……………………2  3. Wolayta…………………3  4. Other, specify------- |  |  |
| 105 | What is your occupation? | 1. House wife ………………1  2. Gov’t employee…………..2  4. Merchant………………….3  7. Other, Specify----- |  |  |
| 106 | How much was your family average annual income in ETB? | annual income------------Birr |  |  |
| 107 | What is your marital status at time of insertion? | 1. Married ……………1  2. Divorced .................2  3. Widowed.................3  4. Never married ........4  5. Other specify------ |  |  |
| 108 | What is the highest grade your husband completed? | 1. Cannot read and write......1  2. Read and write. (0-4 grade).2  3.5-8 grade.............................3  4.9-12 grade..........................4  5. College diploma and above..5 |  |  |
| 109 | What is your partner occupation? | 1. Farmer……….1  2. Merchant ……2  3. Day labor……..3  4. Gov’t employee…4  6. Other, Specify------------- |  |  |
| 110 | Total members of House Hold | _____________person |  |  |

**Part Two; History of contraceptive methods Utilization**

| **S.No** | Questions | Alternative /Choice of response | Code | Skip |
| --- | --- | --- | --- | --- |
| 111 | Have you ever heard of any contraceptive methods? | 1.---------------Yes  2----------------.No |  |  |
| 112 | If yes for Q111, which kind of contraceptive method have you ever heard? (You can answer more than one). | Pills ……………………… 1  IUCD……………………… 2  Inject able………………… 3  Implants ………………….. 4  Male condom………………5  Others (specify)…………… |  |  |
| 113 | From where do you obtain /heard the information? | Health professionals…1  Mass media…………….2  Others (Specify)………… |  |  |
| 114 | Have you ever used any contraception before using Implanon? | 1.------------Yes  2.------------ No |  |  |
| 115 | If yes for Q 205, Which method does you used? (the last method) | Pills……………………………1  IUCD …………………………2  Inject able...…………………..3  Others (specify)…………… |  |  |
| 116 | Date of implanon insertion | ______/_____/______ |  |  |
| 117 | Your Implanon use accepted by your partner? | 1. yes  2. no |  |  |

**Part Three; Past Gynecological and Reproductive information**

| S.No | Questions | Alternative /Choice of response | Code | Skip |
| --- | --- | --- | --- | --- |
| 118 | Do you have children during insertion of implanon? | Yes………………1  No……………….2 |  |  |
| 119 | If yes how many children do you have? | ………………….. |  |  |
| 120 | What is sex difference of children | M---------- F ……… |  |  |

**Part four: Role of Partner and Counseling services**

| 121 | Did you get counseling service before inserting the implanon? | 1…………..Yes  2…………...No |  |  |
| --- | --- | --- | --- | --- |
| 122 | If yes what type of counseling did you obtain? | Individual counseling…….1  Mass counseling …. …2  With husband together….. 3  Other (specify) ………… |  |  |
| 123 | Did you get follow up counseling service after insertion of implanon? | Yes ………1  No…………2 |  |  |
| 124 | Did you first discuss with your partner exactly to use Implanon? | 1…………Yes  2…………No |  |  |
| 125 | Who was decided inserting the Implanon? | 1. my own decision  2. professional decision |  |  |
| 126 | Distance from Health center?(Km) | ……………………km |  |  |
| 127 | Did you feel any side effect after inserting implanon? | 1………….yes  2…………..no |  |  |
| 128 | If yes for Q415, what type of side effect(s)? | 1. menses disturbance…….…..1  2. weight loss/gain………….….2  3. Head ache, acne, dizziness etc..3  4. others, (specify)………………4 |  |  |
| 129 | After the insertion of implanon, did they appointed you at a specific time? | 1……………………yes  2…………………….no |  |  |
| 130 | Did you satisfied by the service given by the FP service provider during the insertion? | 1…………………yes  2………………….no |  |  |

**Part-five: Outcome measuring variables (Reasons for removal of Implanon use)**

| S.N | Questions | Alternative /Choice of response | Skip |
| --- | --- | --- | --- |
| 131 | Are you still using the implanon? (Confirm implanon at insertion site by palpation) | 1………..yes  2………...no |  |
| 132 | If no for Q131, for how long did you utilize implanon? | ……………………(in month) |  |
| 133 | What was the reason for removing implanon? | 1.Disire to be pregnant….1  2.Due to other illness, drug use….2  3.Religen & culture opposition….3  4.Oposed by partner…………..…4  5. Due to weight gain/Weight Loss5  6. Menstrual disturbance………..6  7. Other………………………….7 |  |
| 134 | Place of removal | Governmental Institution…...1  Privet facilities………………2  Other place specify…………3 |  |

This is the end of the interview.

THANK YOU!
